# Can the dexamethasone intravitreal implant Ozurdex be safely administered in an out-of-operating room setting?

**DOI:** 10.1080/21556660.2020.1742723

**Published:** 2020-03-31

**Authors:** María del Pino Cidad-Betegón, Félix Armadá-Maresca, Gloria Amorena-Santesteban, Javier Coca-Robinot, Oriana D'Anna-Mardero, Irene de la Rosa-Pérez, Beatriz Manzano-Muñoz, Jesús García-Martínez, Mónica Asencio-Durán, Gema Casado-Abad

**Affiliations:** a Ophthalmology Department, La Paz University Hospital, Madrid, Spain; b Hospital Pharmacy Service, La Paz University Hospital, Madrid, Spain

**Keywords:** Dexamethasone implant, retina, controlled environment surgical cabin, ArcSterile, safety

## Abstract

**Purpose:**

To describe a standardized protocol of the dexamethasone intravitreal (DEX) implant Ozurdex (Allergan, Dublin, Ireland) performed in a controlled environment surgical cabin (CESC).

**Methods:**

Retrospective and observational study conducted on patients who underwent a DEX implant between May 2011 and June 2019, in a third level University Hospital. The controlled environment surgical cabin (ArcSterile, Imex, Valencia, Spain) used in this study was the MB 20 (2 m width, 1.60 m depth, and 2 m height) with an uninterrupted power system (ARSSAI1) to keep the cabin working for 20 min. The cabin was used in the open mode. A standardized protocol of intravitreal injections in controlled environment surgical cabin was designed.

**Results:**

From May 2011 to February 2015, a total of 454 DEX implants were performed in the operating room, whereas from March 2015 to June 2019, 1054 DEX devices were implanted using the CESC. The mean number of DEX implants/per week was significantly lower in the operating room than in the CESC [2.3 (2.1 to 2.5) versus 3.8 (3.6 to 4.1), mean difference 1.5 (1.2 to 1.8), *p* < 0.0001]. The incidence of endophthalmitis was similar in the two populations, 0/454 (0.0%; 95% CI 0.0 to 0.81%) and 0/1054 (0.0%; 95% CI 0.0 to 0.35%) in the operating room and in the CESC, respectively.

**Conclusions:**

The CESC may be a good alternative to the conventional operating room for the administration of the intravitreal DEX implant.

## Introduction

Diabetic retinopathy (DR) and retinal vein occlusion (RVO) are two leading causes of visual impairment and blindness^1^^,^^2^. Macular edema (ME) has been identified as the most common cause of vision loss in patients affected by DR^2^^,^^3^ and RVO^4^^,^^5^.

Among currently available treatment options, intravitreal injections, either anti-vascular endothelial growth factor (VEGF) or intravitreal corticosteroids, have become first-choice therapy for DME over the past several years^6^^,^^7^.

Despite the good functional and anatomical outcomes obtained with the anti-VEGF therapies, many patients do not adequately respond^8–11^. The question of whether patients who do not adequately respond to anti-VEGF could benefit from an early change to another therapy has not been fully elucidated. Nevertheless, there is new evidence supporting an early switch to DEX in those patients who did not adequately respond to anti-VEGF^12–14^.

Dexamethasone intravitreal (DEX) implant has shown to be an effective treatment for DME in clinical and real-life studies^15–20^.

The question of whether the DEX implant (Ozurdex^®^; Allergan, Irvine, CA) may be safely administered outside the operating room remains^21^. While in many countries, intravitreal injections are performed at the operating room^22^, in the USA and Canada are mainly performed in the office^23^. The Vitreo-Retina Spanish Society (SERV) guidelines did not establish any recommendation about the best place for performing the procedure, i.e. office setting, procedure room, operating room, etc.^24^.

DEX implants should be administered in a space that present enough comfort, both for the patient and for the ophthalmologist, and likewise allow the realization of a sterile technique^21–26^.

The results of a retrospective and observational study, carried out in Spain, which compared the profitability of the controlled environment surgical cabin (CESC) versus the operating room in ophthalmic minor surgical procedures, found that the use of the CESC was associated with an increase of 14% in the number of surgeries^27^. Additionally, the cost per hour of the CESC was 30.75€, while the cost per hour of the conventional operating room was 142.78€, which meant a reduction of the 78.5% in the cost per hour^27^.

This paper aimed to describe a standardized protocol of the DEX implant performed in a controlled environment surgical cabin “The ArcSterile^®^” and its safety. Additionally, this study also compared the number of DEX implants performed in the operating room with those performed in the CESC.

## Methods

Retrospective analysis of a register database of patients who underwent a DEX implant between May 2011 and June 2019, in a third level University Hospital. All the data were collected from the Hospital register database which included patient identification number, age, disease, type of procedure, and serious adverse events.

The study protocol was approved by the ethics committee of La Paz University Hospital (Protocol number HULP: PI-3797), that waived the need of informed consent for this study. All procedures were carried out in accordance with the tenets of the Declaration of Helsinki.

### The ArcSterile

The controlled environment surgical cabin (ArcSterile^®^, Imex, Valencia, Spain) used in this study was the MB 20 (2 m width, 1.60 m depth, and 2 m height) with an uninterrupted power system (ARSSAI1) to keep the cabin working during 20 min^28^. The ArcSterile^®^ is an aluminum structure cabin, with sliding screens panel and folding front opening polyvinyl door for entry of patients. It recreates the conditions of asepsis and electrical safety of a conventional operating room, allowing the realization of invasive procedures in a safe way, in any hospital room (as long as it has an air-conditioned system)^28^.

This device was designed for those processes in which it is crucial to have a low level of particles and microorganisms suspended in the air. The ArcSterile^®^ can act as a chamber of controlled indoor air quality for both, surgical procedures and those that require aseptically special conditions, as isolation rooms^28^.

Regarding its technical design, it includes two columns of impulsion and air filtration to generate the sterile horizontal laminar flow. This device ensures ISO 5 air quality in the operative field^29^, throughout the duration of the surgical process. The level of suspended particles in the air is kept low due to: (1) the ultrafiltration of the air; (2) the laminated characteristics of the air flow; (3) the air renewal; and (4) the positive pressure generated, which prevents outside air penetrate inside the cabin^28^^,^^29^.

#### Mode of operation

Although the ArcSterile^®^ can be placed in any Hospital room, the room should be previously equipped with an air-conditioned system. The laminar air flow tunnel can be switched to be generated from left to right or vice versa, to fit the work organization of the surgical team and the instrumental location.

The cabin could be used in the “open mode” or in the “close mode”. In this protocol, the cabin was used in the open mode.

### Protocol of intravitreal injection pathway

A standardized protocol of intravitreal injections in CESC has been designed. This protocol defines step by step how to optimize the path from the medical retina office, when the doctor decides to set up a new intravitreal injection, until the patient leaves the CESC after the treatment^30^.

The work protocol is shown in Table 1.

**Table 1.** Work protocol for intravitreal injections in controlled environment surgical cabin (ArcSterile^®^).

Protocol
Intervention Staff: One Ophthalmologist One nurse One nurse assistant (recommended) Review of patient medical history and procedure to be performed. The nurse assistant calls the patient who is in the waiting room, puts on the dressing gown and cap. Patient checklist: To verify that the patient has signed the written informed consent to the procedure. Procedure to be performed. Eye to be treated. Out of the surgery cabin, three minutes before procedure starts, topical anesthetic (5% lidocaine) and 5% povidone iodine are applied on the surface of the eye. Once the patient is placed inside the surgery cabin, 5% lidocaine and 5% povidone iodine are applied topical and the skin is painted with 10% povidone iodine. A sterile pack per patient will be used. With the help of sterile gloves (and always with sterile mask) it is placed a sterile fenestrated cloth and an eyelid speculum over the eye to be treated. 5% lidocaine and 5% povidone iodine are applied again on the surface of the eye. Proceed to the intraocular injection previous marked with compass.^a^ Mobilization of the conjunctiva with a surgical microsponge, injection, and reposition of the conjunctiva. Removal of eyelid speculum and sterile fenestrated cloth. Once the intervention is concluded, the ophthalmologist will indicate to the patient the postoperative care, which will be written in a report, and when to request the appointment for re-evaluation in the office

^a^ Dexamethasone intravitreal implant (Ozurdex^®^) 0.7 was placed into the vitreous cavity following standard indications^31^.

The protocol of intravitreal injection pathway in the conventional operating room is similar to that of the CESC. The main difference falls to the intervention staff. In the CESC protocol were involved one ophthalmologist, one nurse, and one nurse assistant (recommended), whereas in the conventional operating room were involved the staff of the operating room (one ophthalmologist, one nurse, one nurse assistant, and one orderly) and those of the ophthalmology outpatient care (one nurse and one nurse assistant) (Table 1).

### Statistical analysis

A standard statistical analysis was performed using MedCalc Statistical Software version 19.0.3 (MedCalc Software bvba, Ostend, Belgium; https://www.medcalc.org; 2019).

Data were evaluated in a masked fashion. The groups were coded and the statistician did not know where the procedure was performed.

Descriptive statistics mean [standard deviation (SD)], mean [95% confidence interval (95% CI)], median (25–75 quartile range), and percentages were used, as needed.

Data were tested for normal distribution using a D’Agostino-Pearson test.

The two-way independent sample *t* Student test was used to compare the number of DEX implant/per week between the operating room and the CESC.

Categorical variables were compared using a Chi-square test and a Fisher's exact test, as needed.

## Results

**Figure 1.**
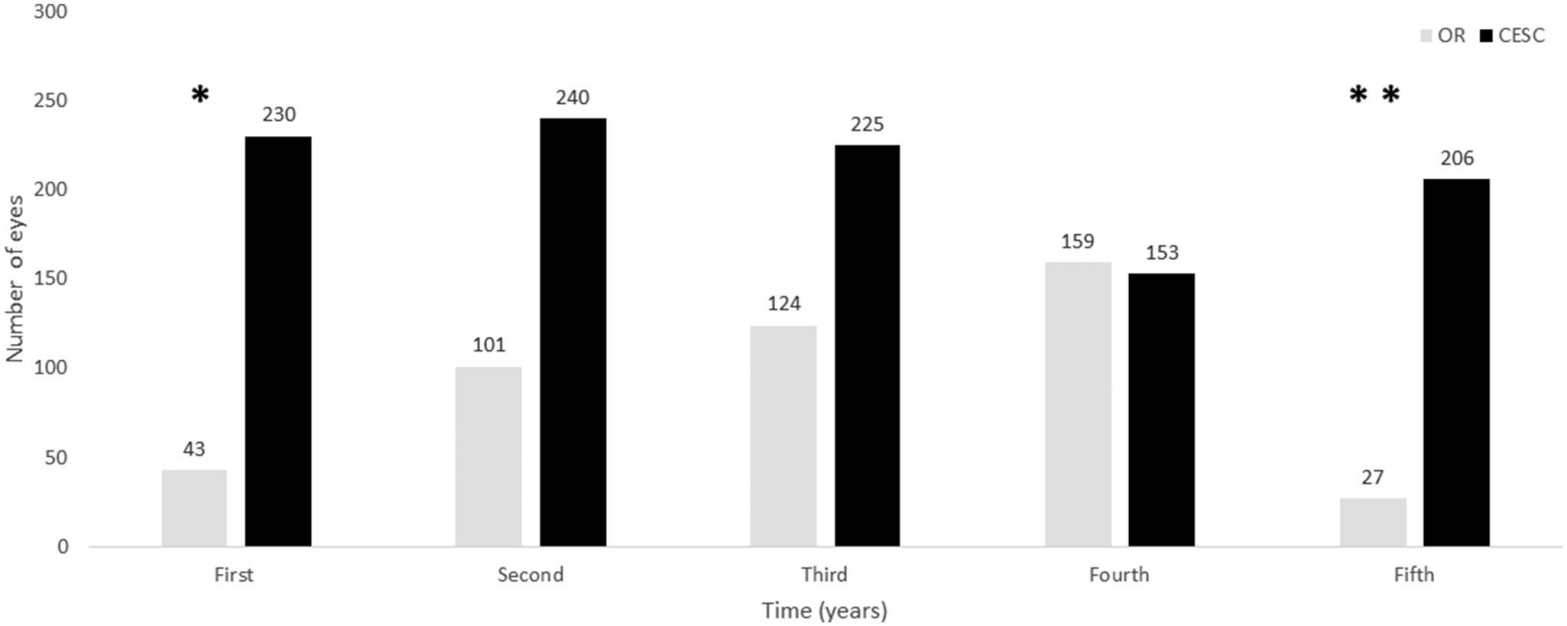
Number of eyes treated with the intravitreal dexamethasone implant Ozurdex. Abbreviations. CESC: controlled environment surgical cabin; OR: operating room. First: Years 2011 and 2015 for the OR and CESC, respectively. Second: Years 2012 and 2016 for the OR and CESC, respectively. Third: Years 2013 and 2017 for the OR and CESC, respectively. Fourth: Years 2014 and 2018 for the OR and CESC, respectively. Fifth: Years 2015 and 2019 for the OR and CESC, respectively. *From 10 May 2011 in the OR and from 4 March 2015 in the CESC. **Until 19 February 2015 in the OR and till 25 June 2019 in the CESC.

From 10 May 2011 to 19 February 2015, a total of 454 DEX implants were performed in the operating room, whereas from 10 March 2015 to 25 June 2019, 1054 DEX devices were implanted using the CESC (Figure 1). In other words, over the course of the study, in the operating room were performed, on average (95% confidence interval, 95% CI), 2.3 (2.1 to 2.5) DEX implants per week, while in the CESC were performed 3.8 (3.6 to 4.1) DEX implants per week, mean difference 1.5 (1.2 to 1.8), *p* < .0001.

Mean (95% CI) age was 69.4 (68.2 to 70.5) years and 75.1 (74.0 to 76.2 years) for patients underwent DEX implant in the conventional operating room and in the CESC, respectively, *p* < 0.0001.

The incidence of endophthalmitis was similar in the two populations, 0/454 (0.0%; 95% CI 0.0 to 0.81%) and 0/1054 (0.0%; 95% CI 0.0 to 0.35%) in the operating room and in the CESC, respectively.

Accepting an alfa of 0.05 (bilateral hypothesis contrast), with 454 patients in the operating room group and 1054 patients in the controlled environment surgical cabin group, this analysis had a statistical power of the 75% to detect, as statistically significant, an endophthalmitis incidence of the 0.04% in the operating room group and the 0.075% in the CESC group.

With the exception of one eye, performed in the operating room, that received a DEX implant into the crystalline lens, there were not register serious adverse events. The incidence of minor adverse events was not collected.

## Discussion

The CESC ArcSterile^®^ is a tool that speeds up the entire process and allows to handle large volumes of patients with the necessary sterility guarantees^27^.

A CESC has been available in our Department since February 2015. As the results of the current study clearly suggested, its introduction in the clinical management of DEX implant administration has entailed a significant increase in the number of procedures, without a loss of the safety conditions. However, it cannot be ruled out that the increase in the number of DEX implants after the introduction of the CESC device was not related to the approval of DEX implant in the EEUU and most of the European countries for the treatment of DME in 2014^32^. The Spanish National Health System (SNHS) is public, universal, and mostly free of charge for the patients except for the share of out-of-pocket expenditure^33^. All the different treatment options currently available for DME are covered by the SNHS^33^. Since there was not any change in the SNHS regulation, reimbursement of the DEX implant was not the reason of the increase in the number of procedures.

As a consequence of economic, social, and demographic changes, with the resulting implications for health care costs, increasing the efficiency and efficacy of health services became relevant to enable their greater profitability^34^^,^^35^.

Intravitreal injection is a daily practice in Retina Subspecialty^22^^,^^36^. Moreover, the introduction of new intravitreal therapies predicts a future with a growing volume in the number of processes, which will suppose that the Health Systems will have to face the challenge of handling increased patient volume^22^. This fact forces us to create protocols and clinical pathways that allow us to manage a huge number of patients.

In this aspect, the CESC has shown to be effective for treating a greater number of patients and, additionally, to treat them earlier than with the conventional operating room^27^. Our protocol did not significantly differ from that of the European Guidelines^22^.

It was previously suggested that the CESC has a positive economic impact of the health system^27^. However, that variable was not analyzed in our study. Operating room time is a limited commodity, which should be optimized for those procedures that really need it. Administering intravitreal injections at the operating room would increase time per patient treated, running costs, and overall inconvenience to the patient^8^^,^^27^.

Patients underwent DEX implant in the CESC were significantly older than those performed in the operating room. In the one hand, this finding indicated that the DEX implant are administered to an older population. On the other hand, it also suggested that DEX implant administration can be safely performed in elderly people in a CESC.

Regarding the safety profile, the incidence of endophthalmitis did not significantly differ from those reported in the literature^37–41^. Based on the currently available scientific evidence, intravitreal injections performed at the operating room did not have lower rates of endophthalmitis than those performed in the office^21–23^^,^^37–41^.

The incidence of endophthalmitis after intravitreal injections of anti-VEGF or corticosteroids is low^37–41^. In a Retrospective, nationwide multicenter case series conducted in France, which evaluated 316,576 intravitreal injections, the overall incidence of endophthalmitis was 0.021% (2.1 in 10,000 injections) (95% CI, 0.016%−0.026%)^37^. Interestingly, VanderBeek et al. reported a higher risk of endophthalmitis after intravitreal corticosteroids (rate = 0.13% or 1/778 steroid injections) than after anti-VEGF (rate = 0.019% or 1/5283 anti-VEGF injections)^40^. However, the incidence of endophthalmitis in the corticosteroid group was much higher than that observed in other studies^37–39^^,^^41^. Although the number of injections in our study was much lower than that analyzed by other authors^37–41^, the incidence of endophthalmitis observed (95% CI 0.0 to 0.35%) was in line with those previously reported^37–41^.

Last but not least, some comments should be made about DME diagnosis. Although DME can be detected by stereoscopic slit-lamp examination using a fundus lens, the importance of optical coherence tomography (OCT) in diagnosing and managing DME needs to be emphasized^42^.

OCT was originally developed using time-domain acquisition of images^43^. New OCT devices using spectral-domain acquisition of images (SD-OCT) and swept-source OCT (SS-OCT) have been developed. SD-OCT and SS-OCT provide faster acquisition of images, denser sampling of the macula, and better imaging of the choroid and outer retina^44–47^.

However, due to they use different segmentation algorithms for defining retinal layers, normal value for SD-OCT and SS-OCT differ, and measurements are not interconvertible across instruments made by different companies^45^^,^^46^^,^^48^.

This study has some limitations that should be taken into consideration when interpreting its results. First, its retrospective design. Bias and potential pitfalls are inherent to retrospective studies. Second, the results were obtained from a register database with limited information, which supposed that nonserious adverse events could not be analyzed. Third, it was a single-center study that adopted a new protocol and clinical pathway, so our results may be not exportable to other Hospitals. The fourth limitation is the lack of sample size calculation before to start the study. Although it was not originally planned, we have calculated the statistical power for the endophthalmitis rate comparison. We are aware that this was post-hoc analysis; nevertheless, it may provide a valuable information.

Despite these limitations, the controlled environment surgical cabin ArcSterile^®^ may be a good alternative to the conventional operating room for the administration of the intravitreal dexamethasone implant Ozurdex^®^. The CESC is a tool that dynamizes the entire process and can handle large volumes of patients with the necessary guarantees of sterility. Finally, the CESC might allow to reserve the conventional operating room for patients who need a more complex surgery, optimizing the use of operating room

Further studies are needed, especially multicenter prospective trials, that evaluate the role of the CESC on clinical outcomes and health economics.

## Transparency

### Declaration of financial/other relationships

MdPCB has received a grant from Allergan during the conduct of the study. None of the coauthors or peer reviewers of this manuscript have any competing interests to declare.
